# Implementation of a structured practical activity to analyse student healthcare worker perceptions and compliance with prescribed infection control procedures

**DOI:** 10.1186/s12909-021-03048-1

**Published:** 2021-12-14

**Authors:** Elise S. Pelzer, Zachary Stewart, Holly Peters, Jessica O’Callaghan, Emily Bryan, Lucas Wager, Juliana Chiruta

**Affiliations:** 1 Queensland University of Technology, School of Biomedical Sciences, Faculty of Health, 2 George Street, Brisbane City, QLD 4000 Australia; 2 Queensland University of Technology, PO Box 2434, Brisbane, Queensland 4001 Australia

**Keywords:** Healthcare worker, Undergraduate students, Infection control

## Abstract

**Background:**

Non-compliance with infection control guidelines has been reported within healthcare settings. Infection control education in undergraduate healthcare education programs forms a critical component in preparing student healthcare workers for vocational roles.

**Methods:**

Clinical sciences students (nutrition science, paramedicine, pharmacy, podiatry, optometry studying for qualifications recognised by the Australian Health Practitioner Regulation Agency) self-reported hygiene perceptions and practices and collected microbiological swabs from personal or medical equipment items before and after recommended disinfection procedures.

**Results:**

Cultivable microorganisms were isolated from 95% of student medical equipment items. Disinfection significantly reduced microbial growth on student medical equipment items (*P* < 0.05).

**Conclusions:**

Student perceptions of infection control procedures do not always correlate with infection control practice. Infection control education of undergraduate healthcare students requires ongoing assessment to ensure successful translation into clinical practice.

**Supplementary Information:**

The online version contains supplementary material available at 10.1186/s12909-021-03048-1.

## Background

Breaking the chain of infection underpins infection prevention and control policy, however, teaching this content to undergraduate students studying health courses is challenging, with infection control frequently seen as boring. Concerningly, healthcare-associated infections (HAIs) are the most common preventable condition affecting hospitalized patients [1]. Each year in Australia, some 180,000 patients suffer HAIs that increase patient morbidity and mortality risks, resulting in prolonged hospitalization, reduced quality of life, and increased healthcare costs for both patients and the healthcare system [1]. Frequent-touch personal and medical equipment used by healthcare workers in performing their duties is a well-accepted vector for the transmission of microorganisms including drug resistant pathogens, which are capable of surviving on fomites for a period of hours to months, but are invisible to the naked eye [7]. Research indicates that contamination of clothing and small medical equipment items including stethoscopes and pen lights is substantial, and is equivalent to that observed on the hands of healthcare workers (HCW) [9]. Knowledge of the ability of clothing and medical items to act as vectors for carriage of transient microorganisms is critical for reinforcing HCW understanding of procedures that can interrupt the chain of infection [12].

Infection prevention and control practices aim to control and reduce the risk of HAI transmission by breaking the chain of infection between the microbe and the susceptible host: HCW, fomites, and patients. Regulatory bodies, including The Centers for Disease Control and Prevention (CDC) and The National Health and Medical Research Council (NHMRC), provide infection control guidelines for healthcare workers [11]. Allied health boards subscribe to these same infection control guidelines, supporting the need for inclusion of opportunities to embed these guidelines in undergraduate clinical sciences teaching programs (Australian Health Practitioner Regulation Agency). However, evidence suggests that HCWs demonstrate poor compliance with infection control protocols, potentially as a result of limited infectious disease knowledge [2, 3].

Understanding and implementing effective and safe infection control begins at the training level when student healthcare workers undertake their vocational training at educational institutions [13, 14]. Previous studies report a suboptimal commitment to cleaning and disinfection of personal and medical equipment items by both clinical sciences students and experienced HCWs [6]. The aim of this study was to assess the microbial load on undergraduate clinical sciences (nutrition science, paramedicine, pharmacy, podiatry, optometry studying for qualifications recognised by the Australian Health Practitioner Regulation Agency) student’s personal or medical equipment items to: (1) create a tangible inquiry based learning opportunity regarding the presence of “invisible” microorganisms and infectious disease transmission routes; (2) determine whether student hygiene perceptions and practice matched those prescribed by NHMRC prevention and control of infectious disease guidelines; and (3) leverage the results to highlight the efficacy of a simple disinfection procedure on reducing the risk of infectious disease transmission.

## Methods

### Participants

Undergraduate students enrolled in second- or third-year clinical science (nutrition science, paramedicine, pharmacy, podiatry, optometry studying for qualifications recognised by the Australian Health Practitioner Regulation Agency) units undertaking a mandatory single microbiology focused infection control unit.

### Data collection and analysis

Data collection for this pilot study employed convenience sampling, whereby all students enrolled in the mandatory infection control units were invited to participate in the research project. Students were required to complete a survey specifically developed for this study that was administered prior to laboratory work. The survey settings used a combination of short response questions and questions assessed using a 5-point Likert scale; these questions were designed to obtain data relating to students’ self-reported infection control practice (frequency of compliance with prescribed infection control guidelines) and perceived importance of infection control practices. Students were asked to rate their answers from 1 (strongly disagree) to 5 (strongly agree).

Data analysis involved relating student self-reported infection control practices and disinfection procedure/s for personal or medical equipment with the microbiological test culture results obtained from sampling the item during the laboratory classes. Personal items included clothing and personal protective equipment described in the literature as a transmission source of transient microorganisms and medical equipment included items used to perform medical assessments on patients where the student had already participated in clinical placement rotations. As part of the survey, students created a unique code for their data set to de-identify the data. Student creation of the code ensured that the research staff remained blinded to the identity of all student samples. The codes were used by research staff for the purpose of matching survey data and samples for data analysis.

### Microbiology screening

Students were encouraged to select relevant personal or medical equipment items independently due to constraints surrounding the diverse student cohort, discipline-specific timing of clinical placement, and required medical items. This process ensured inquiry-based student learning opportunities to meet the objective of demonstrating to students that simple and regular disinfection of items (both personal and medical) reduces the risk of transmission of potential pathogens to themselves and their patients. Specimens for microbiological screening of students’ personal or medical equipment items were collected by students aseptically using sterile swabs moistened in sterile distilled water (Edwards, Narellan, New South Wales). Specimens were inoculated onto Columbia Horse Blood Agar Plates (Edwards) and incubated for 18–24 h at 37 °C in air. All inoculated plates were examined for microbial growth, the number of colony-forming units (CFU) where growth was present, and colonial morphology were recorded.

### Statistical analysis

All statistical tests were performed using R 3.4.2 software [15]. Microbial counts were categorised ordinally to enable statistical tests to be performed, as too numerous to count (TNTC) observations were not capable of being converted into continuous values. Categories correspond to whole integers on a log10 scale; highest to lowest of ordinal scale = TNTC > 101–340 CFU > 11–100 CFU > 1–10 CFU > 0 CFU (no growth). Microbial diversity was measured as the number of morphologically distinctive colony types visually evident on cultured media.

Wilcoxon signed rank test, with continuity correction, comparing paired results before and after treatment was performed to ascertain whether the in-class treatment was effective for reducing the number of viable microbes on each student’s medical item. The proportion of items with reduced growth after treatment, i.e. colony counts, were categorised into a lower growth category after treatment was calculated.

Additional Wilcoxon signed rank test with continuity correction analysis was performed with paired results, before and after treatment, to determine whether in-class treatment reduced the diversity of cultivable microbes on each student’s medical item.

Additional analysis was completed to determine whether the medical items of students who provided certain answers to the quiz questions were more likely to have higher cultured microbial counts or diversity than others. Kruskal-Wallis rank sum tests were used to test this hypothesis, with subsequent utilisation of Dunn’s test of multiple comparisons to further investigate any significant results, to identify which quiz responses were correlated to microbial growth or diversity results.

## Results

A total of 93 student data sets (survey responses combined with microbiology culture data) were available for analysis. Identifiable datasets were excluded from analysis.

### Microbiological investigations

Medical and personal items screened by undergraduate clinical sciences students included: stethoscopes (36.6%), pens (15.1%, which included responses such as “marker pen”, “biro”, “ink pen”, and “writing pen”), mobile phones (11.8%), pen lights (7.5%), safety glasses (7.5%), glasses (5.4%, corrective eyewear), clothes (4.3%), scissors (3.2% scissors and trauma sheers), and other (8.6%, such as laptop) (Table 1).

**Table 1:** Students’ personal or medical equipment items

Medical equipment item	Number of items	%
Stethoscope	34	36.6
Pen	14	15.1
Mobile phone	11	11.8
Other	8	8.6
Pen light	7	7.5
Safety glasses	7	7.5
Glasses	5	5.4
Clothes	4	4.3
Scissors	3	3.2
Total	93	100.0

Cultivable microorganisms were isolated from 94.7% of personal or medical equipment items prior to in-class disinfection and from 46.2% of medical equipment items after in-class disinfection (Table 2). In-class disinfection significantly reduced the number of CFUs cultured from medical equipment items for 80.7% of medical items (*P* < 0.0001).

**Table 2:** Microbial load (CFU) on medical equipment item

Medical equipment item (number of items)	TNTC		101–340		11–100		1–10		No growth	
	Before	After	Before	After	Before	After	Before	After	Before	After
Clothes (4)	2	1	0	0	1	1	1	0	0	2
Mobile phone (11)	1	0	2	1	6	1	1	3	1	6
Pen (14)	0	0	0	0	9	0	4	6	1	8
Stethoscope (34)	5	0	2	0	14	2	13	13	0	19
Scissors (3)	0	0	1	0	0	0	1	2	1	1
Safety glasses (7)	4	0	0	0	2	0	0	5	1	2
Glasses (5)	3	0	1	0	0	0	1	2	0	3
Pen light (7)	0	0	0	0	2	0	4	2	1	5
Other (8)	1	0	1	0	3	1	3	3	0	4
**Total (93)**	**16**	**1**	**7**	**1**	**37**	**5**	**28**	**36**	**5**	**50**
%	17.2	1.1**	7.5	1.1*	39.8	5.4***	30.1	38.7	5.4	53.8

*CFU* colony forming units, *TNTC* too numerous to count

*P < 0.05, ** *P* < 0.001, ***P < 0.0001

**Fig. 1:**
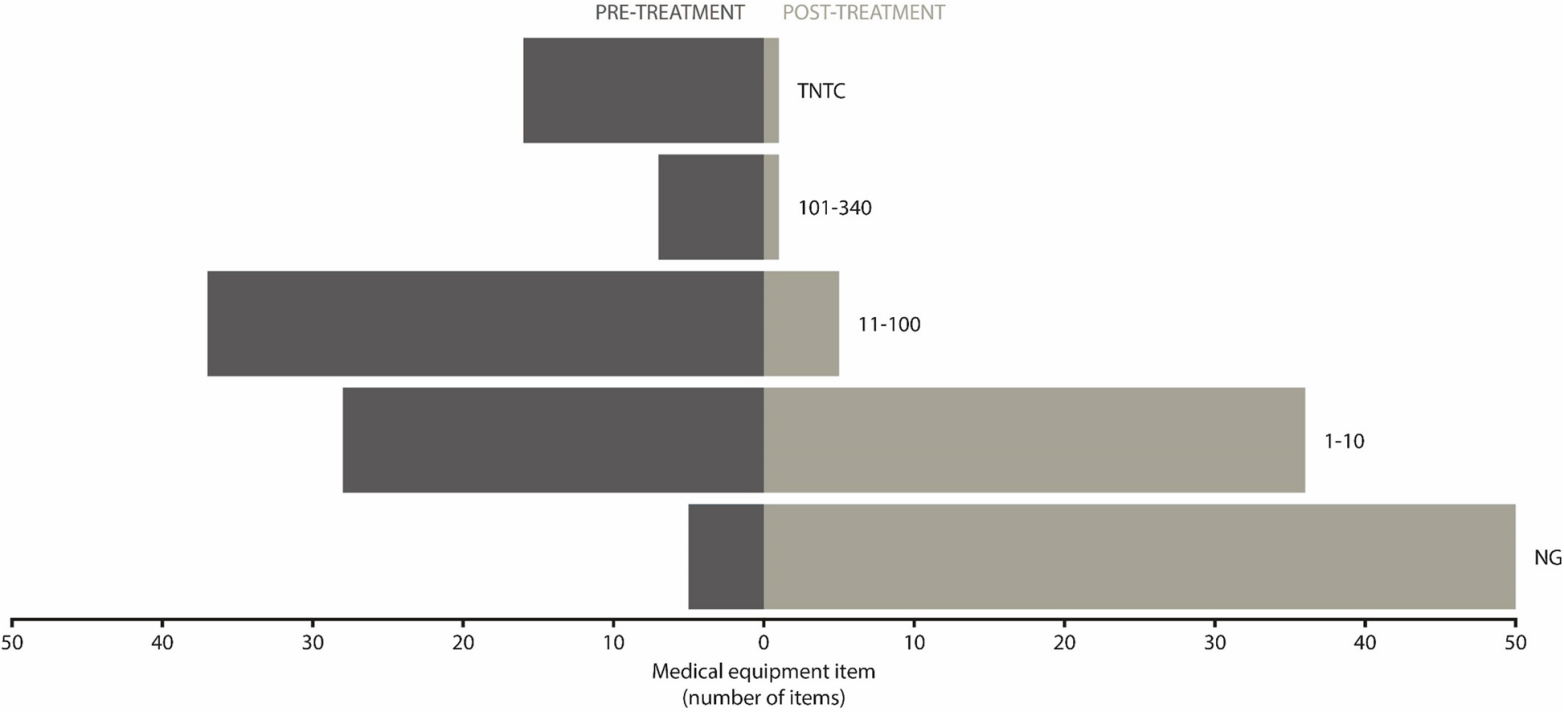
Colony forming units on student’s personal or medical equipment items pre- and post-disinfection. Cleaning personal medical equipment reduces the titre of cultivatable bacteria. Student’s personal or medical equipment items were swabbed pre- and post-disinfection. Columbia horse blood agar plates were inoculated with these swabs, incubated for 18–24 h at 37 °C and the CFUs were counted. CFU counts were binned into the categories: TNTC > 101–340, CFU > 11–100, CFU > 1–10, CFU < 1–10 (no growth; NG) and a two-sided frequency histogram plotted of the results

Viable counts in the ranges of TNTC, 101–340 CFU and 11–100 CFU, from swabs of personal or medical equipment items were significantly decreased after disinfection when compared to before disinfection (*P* = 0.0002, *P* = 0.03 and *P* < 0.0001 respectively). Increased colony counts were observed for the majority of culture plates with initial viable counts of 1–10 CFU or no growth recorded prior to disinfection, indicating that the items were re-contaminated (Table 2, Fig. 1).

Moreover, results from the same test showed that in-class treatment significantly reduced the diversity of CFU cultured on agar after treatment, of which 73.1% of medical items evinced reduced cultured diversity (morphotypes), when compared to the cultured diversity obtained before treatment (*P* < 0.0001, data not shown).

### Survey data

The majority of all respondents agreed or strongly agreed that infection control was critical for protecting self and patients or clients from infectious disease (85.0%), although more students strongly agreed that it was critical for patients or clients (69.9%) rather than for protection of self (58.1%) (Table 3). Medical item disinfection frequency was low, with the majority of students (53.8%) admitting that they never disinfected their chosen medical or personal item. Only four students (4.3% of total) followed proper clinical practice of disinfecting their item after each patient. For students who did elect to clean their item at any frequency, most respondents (30.1%) used a 70% (v/v) ethanol or isopropanol cleaning agent rather than water only (5.4%), soap and water (5.4%), or some “other” disinfectant-grade cleaning agent (8.6%). Students were slightly more likely to clean medical items in direct contact with patients (43.0%) or themselves (41.9%) and less frequently for items in indirect contact with patients (57.0%).

**Table 3:** Infection control perceptions and practices of student HCWs

Variable	n =	%
***Frequency of medical item disinfection***		
*Never*	50	53.8%
*Once a year*	10	10.8%
*Once a week*	12	12.9%
*Daily*	6	6.5%
*After each patient*	4	4.3%
*Other*	11	11.8%
***Agents used for cleaning medical equipment***		
*No response*	47	50.5%
*Water only*	5	5.4%
*Soap and water*	5	5.4%
*70% ethanol/isopropanol*	28	30.1%
*Other*	8	8.6%
***Frequency of medical equipment item disinfection of components in*** ***direct*** ***contact with patients***		
*Never*	40	43.0%
*Once a year*	6	6.5%
*Once a week*	11	11.8%
*Daily*	9	9.7%
*After each patient*	20	21.5%
*Other*	7	7.5%
***Frequency of medical equipment item disinfection of components in*** ***indirect*** ***contact with patients***		
*Never*	53	57.0%
*Once a year*	5	5.4%
*Once a week*	19	20.4%
*Daily*	7	7.5%
*After each patient*	3	3.2%
*Other*	6	6.5%
***Frequency of medical equipment item disinfection of components in direct contact with myself***		
*Never*	39	41.9%
*Once a year*	5	5.4%
*Once a week*	19	20.4%
*Daily*	10	10.8%
*After each patient*	11	11.8%
*Other*	9	9.7%
***Infection control practice is critical to protecting me from infectious diseases***		
*Strongly disagree*	13	14.0%
*Neutral*	1	1.1%
*Agree*	25	26.9%
*Strongly agree*	54	58.1%
***Infection control practice is critical to protecting my patients/clients from infectious diseases***		
*Strongly disagree*	14	15.1%
*Agree*	14	15.1%
*Strongly agree*	65	69.9%

### Correlation between routine disinfection practice and microbial load

Whilst most items screened would be considered frequent-touch items, certain personal and medical items were more and others less likely to result in the recovery of an increased number of CFUs (*P* = 0.0323). The number of viable CFUs was independent of student perceptions and infection control practices (Supplementary Table 1). Microbial diversity was not significantly correlated with any quiz question response (Supplementary Table 2). Medical equipment items comprised predominantly of non-porous materials, including metals and plastics (pen, pen light, and scissors), could be considered the cleanest items, while glasses (safety glasses and corrective eyewear), clothes, and mobile phones could be considered the dirtiest items (Supplementary Table 1).

## Discussion

Infection prevention and control training in undergraduate HCW courses frequently lacks structured microbiology-based practical activities targeting the chain of infection. HCW student personal and medical equipment items harboured a diverse range of microorganisms, with colony counts often exceeding the normalisation level for clean, classified as less than 20 CFU [12]. Personal and medical equipment items are reported to be potential sources of microbial transmission between HCWs and patients. This project created a unique opportunity to understand the current perceptions and practises of healthcare students and to improve student understanding of the role of infection control practices for safe treatment of patients by providing an opportunity for students to visualise “invisible” microorganisms harboured on items used during patient interactions before and after low-level disinfection. In agreement with previous studies, we confirmed that disinfection significantly reduced the microbial load on personal and medical equipment items. In line with previous reports, alcohol wipes provided an effective method for reducing the microbial load and diversity of microorganisms cultivated from medical and personal equipment items [8, 10, 16].

Whilst it is widely accepted that non-critical medical equipment items are not meant to be sterile, the impact of frequent-touch personal and medical equipment items in the transmission of infectious disease in clinical settings should be actively managed (NHMRC). In this study, students enrolled in undergraduate courses where infection control is paramount to patient health were asked to self-report their own practises, as well as swab a piece of their own equipment, to determine its cleanliness. Our results suggest that certain medical and personal equipment items are more likely to harbour microorganisms than others. Generally, items worn on the person’s body (e.g. clothes and corrective eyewear) or carried around and handled regularly throughout the day (e.g. mobile phones) were most likely to be contaminated with viable microbes, compared to items that are used irregularly or only for short periods of time in a medical context (e.g. pens, pen lights, or scissors), which can be considered to be the “cleanest”. This observation is consistent with previous reports [5]. Although we hypothesised that students who cleaned their items more regularly, or believed that following infection control guidelines was important, would have a reduced microbial load and diversity recovered from their personal medical equipment, our data does not support this. It is possible that belief does not always translate into action, and that for students who do actively clean their equipment, re-contamination occurs rapidly. Combined with our data and the knowledge that re-contamination of equipment after disinfection can occur and is correlated with hand hygiene behaviour in the clinical setting we plan to make more explicit reference to the concept of cross-contamination in future practical activities [9].

Infection control practices can be improved in trainee HCWs. A limitation of this project was that we did not survey our students at the completion of the activity to assess the impact of visualising the microbiological load on personal and medical equipment items pre- and post-disinfection using a standardised survey tool. To fully evaluate this strategy as a learning activity, this aspect should be included in future research. In the absence of a post-activity survey, we analysed anecdotal evidence provided as anonymous feedback on unit learning activities, which highlighted the value and impact of the practical activity for improving infection prevention and control training in undergraduate HCW students (Supplementary Table 3). The potential benefits of delivering infection control activities where the students and their personal or medical equipment items are the subject, increases awareness and opens an avenue for discussing infection control compliance in a low-risk environment. Appropriate infection control practices that prevent the transmission of infectious organisms are critical in reducing the incidence of HAIs. These measures are the shared responsibility of every individual working in or visiting a healthcare facility, including trainee HCWs. The number of preventable HAIs has been reduced as a result of improved campaigns targeting HCW compliance with infection control procedures such as hand hygiene [4, 17]. The data generated by this study has the potential to impact industry practice and improving compliance through increased awareness of deficits in student HCW hygiene behaviours related to personal medical equipment.

## Conclusions

Student perceptions of infection control procedures do not always correlate with infection control practice. This project utilised an inquiry-based approach to enable clinical sciences students to analyse their own personal and medical equipment items as potential vectors for the transmission of infectious diseases. The data generated by this project created an impetus for focus on infection prevention and control as part of quality care, and in our cohort translated to improved risk assessment of infectious disease transmission opportunities and understanding of the invisible world of microbiology, and increased compliance with infection prevention and control strategies such as hand hygiene, and PPE selection and correct donning/doffing procedures over the duration of the unit. Infection control education of undergraduate healthcare students requires the inclusion of practical microbiology activities to ensure successful translation into clinical practice.

## Supplementary Information

**Additional file 1.**

**Additional file 2.**

## Data Availability

The datasets used and/or analysed during this current study are available from the corresponding author on reasonable request.

## Abbreviations

CFU: Colony-forming unit
HAI: Healthcare-associated infection
HCW: Healthcare worker
TNTC: Too numerous to count
