# Supplementary material for: Implementation of a structured practical activity to analyse student healthcare worker perceptions and compliance with prescribed infection control procedures
Source: BMC Med Educ. 2021 Dec 14;21:617. doi: 10.1186/s12909-021-03048-1 (PMC8672573; doi:10.1186/s12909-021-03048-1)
Supplement: Supplementary file 1 — Additional file 1. [file 12909_2021_3048_MOESM1_ESM.docx]

**Supplementary Files**

Implementation of a structured practical activity to analyse student healthcare worker perceptions and compliance with prescribed infection control procedures

**Authors**

Elise S. Pelzer^1,2^* , Zachary Stewart^1^, Holly Peters^1^, Jessica O’Callaghan^1,2^, Emily Bryan^1,2^, Lucas Wager^1^, Juliana Chiruta^1^

^1^ Queensland University of Technology, School of Biomedical Sciences, Faculty of Health, 2 George Street, Brisbane, Queensland, 4000, Australia

^2^ Institute of Health and Biomedical Innovation, Faculty of Health, Queensland University of Technology, Brisbane, Queensland, 4001, Australia

**Corresponding author**

Elise Pelzer

Queensland university of Technology

PO Box 2434

Brisbane, Queensland, Australia 4001

+ 617 3138 0542

[e.pelzer@qut.edu.au](mailto:e.pelzer@qut.edu.au)

**Supplementary File 1:** Questionnaire for perception of infection control practices of undergraduate clinical sciences students

1. What medical equipment item did you screen for microbial contamination? Please specify.
2. I regularly disinfect my personal medical equipment (for example pen lite, stethoscope, PDA).

□ Never

□ Once a year

□ Once a week

□ Daily

□ After each patient

□ Other: please specify __________________________________________________

1. If you routinely disinfect your medical equipment, which cleaning agent do you use?
   □ Water only

□ Soap and water

□ 70% ethanol/isopropanol

□ Other: please specify__________________________________________________

1. I regularly disinfect components of my medical equipment that are in direct contact with patients (for example stethoscope membrane).

□ Never

□ Once a year

□ Once a week

□ Daily

□ After each patient
□ Other: please specify________________________________________________________

1. I regularly disinfect components of my medical equipment that are not in direct contact with patients (for example pen lite).

□ Never

□ Once a year

□ Once a week

□ Daily

□ After each patient
□ Other: please specify________________________________________________________

1. I regularly disinfect parts of my medical equipment that are in direct contact with my person (for example stethoscope ear buds).

□ Never

□ Once a year

□ Once a week

□ Daily

□ After each patient

□ Other: please specify__________________________________________________

1. I believe that infection control practice is critical to protecting me from infectious diseases

□ Strongly disagree

□ Disagree

□ Neutral

□ Agree

□ Strongly agree

1. I believe that infection control practice is critical to protecting my patients/clients from infectious diseases

□ Strongly disagree

□ Disagree

□ Neutral

□ Agree

□ Strongly agree
