# Supplementary material for: Implementation of a structured practical activity to analyse student healthcare worker perceptions and compliance with prescribed infection control procedures
Source: BMC Med Educ. 2021 Dec 14;21:617. doi: 10.1186/s12909-021-03048-1 (PMC8672573; doi:10.1186/s12909-021-03048-1)
Supplement: Supplementary file 2 — Additional file 2. [file 12909_2021_3048_MOESM2_ESM.docx]

**Supplementary Tables**

Implementation of a structured practical activity to analyse student healthcare worker perceptions and compliance with prescribed infection control procedures

**Authors**

Elise S. Pelzer^1,2^* , Zachary Stewart^1^, Holly Peters^1^, Jessica O’Callaghan^1,2^, Emily Bryan^1,2^, Lucas Wager^1^, Juliana Chiruta^1^

^1^ Queensland University of Technology, School of Biomedical Sciences, Faculty of Health, 2 George Street, Brisbane, Queensland, 4000, Australia

^2^ Institute of Health and Biomedical Innovation, Faculty of Health, Queensland University of Technology, Brisbane, Queensland, 4001, Australia

**Corresponding author**

Elise Pelzer

Queensland university of Technology

PO Box 2434

Brisbane, Queensland, Australia 4001

+ 617 3138 0542

[e.pelzer@qut.edu.au](mailto:e.pelzer@qut.edu.au)

**Supplementary Tables**

**Supplementary Table 1.** Kruskal-Wallis rank sum test results comparing the amount of microbial growth (ordinal measure; 0, 1-10, 11-100, 101-340, too numerous to count [TNTC]) against student quiz responses to ascertain whether students who provide certain quiz responses have more or less growth before or after treatment. Where significant differences were found, post-hoc testing using Dunn’s test of multiple comparisons was performed to identify which quiz responses were correlated to microbial growth results. Only significant pairs identified through Dunn’s test are shown here to reduce table size (all pairs not shown have P-value > 0.05). *: ≤ 0.05, **: ≤ 0.01, ***: ≤ 0.001

| **Before** | **Kruskal-Wallis rank sum** | | |  | **Dunn’s test** | | |
| --- | --- | --- | --- | --- | --- | --- | --- |
|  | *Df* | *χ2* | *P-value* | *Group* | | *Z-score* | *P-value* |
| Quiz Question 1 | 7 | 15.30512043 | 0.032281149* | Glasses-pen | | 2.290008 | 0.022021* |
|  |  |  |  | Clothes-pen light | | 2.112951 | 0.034605* |
|  |  |  |  | Glasses-pen light | | 2.881982 | 0.003952** |
|  |  |  |  | Mobile phone-pen light | | 2.017283 | 0.043666* |
|  |  |  |  | Pen-safety glasses | | -2.08696 | 0.036891* |
|  |  |  |  | Pen light-safety glasses | | -2.73239 | 0.006288** |
|  |  |  |  | Glasses-scissors | | 2.006574 | 0.044795* |
| Quiz Question 2 | 5 | 5.85527648 | 0.32055687 | - | |  | - |
| Quiz Question 3 | 3 | 1.98247196 | 0.576052674 | - | |  | - |
| Quiz Question 4 | 5 | 5.925861881 | 0.313499867 | - | |  | - |
| Quiz Question 5 | 5 | 8.072464497 | 0.152286926 | - | |  | - |
| Quiz Question 6 | 5 | 4.515879437 | 0.477761639 | - | |  | - |
| Quiz Question 7 | 3 | 3.218627412 | 0.359129709 | - | |  | - |
| Quiz Question 8 | 2 | 3.649242988 | 0.161278678 | - | |  | - |

| **After** | **Kruskal-Wallis rank sum** | | |  | | **Dunn’s test** | | |  |
| --- | --- | --- | --- | --- | --- | --- | --- | --- | --- |
|  | *Df* | *χ2* | *P-value* | | *Group* | |  | *P-value* | |
| Quiz Question 1 | 7 | 3.804409698 | 0.802009909 | | - | |  | - | |
| Quiz Question 2 | 5 | 4.497671608 | 0.480195029 | | - | |  | - | |
| Quiz Question 3 | 3 | 1.13305384 | 0.769101337 | | - | |  | - | |
| Quiz Question 4 | 5 | 6.728452074 | 0.24163155 | | - | |  | - | |
| Quiz Question 5 | 5 | 4.958958667 | 0.420909461 | | - | |  | - | |
| Quiz Question 6 | 5 | 2.974950948 | 0.703847839 | | - | |  | - | |
| Quiz Question 7 | 3 | 3.996396388 | 0.261853518 | | - | |  | - | |
| Quiz Question 8 | 2 | 2.489263531 | 0.288046957 | | - | |  | - | |

**Supplementary Table 2.** Kruskal-Wallis rank sum test results comparing the amount of microbial diversity i.e., the discrete number of different colony types observed against student quiz responses to ascertain whether students who provide certain quiz responses have more or less microbial diversity before or after treatment. Post-hoc testing was not performed as no significant results were found.

| **Before** | | **Kruskal-Wallis rank sum** | | |
| --- | --- | --- | --- | --- |
|  | *Df* | | *χ2* | *P-value* |
| Quiz Question 1 | 7 | | 12.24529406 | 0.092775357 |
| Quiz Question 2 | 5 | | 4.578588406 | 0.469437928 |
| Quiz Question 3 | 3 | | 3.008557201 | 0.390307705 |
| Quiz Question 4 | 5 | | 5.382137832 | 0.371043018 |
| Quiz Question 5 | 5 | | 0.530677008 | 0.990956916 |
| Quiz Question 6 | 5 | | 7.793260207 | 0.168003552 |
| Quiz Question 7 | 3 | | 1.936387975 | 0.585714016 |
| Quiz Question 8 | 2 | | 2.701067175 | 0.25910197 |

**Supplementary Table 3.** Select examples of anonymous student feedback provided for practical activities.

| **Anonymous student comments** |
| --- |
| Swabbing my equipment really enforced the idea that bacteria are all around us and that it is important for everyone to take steps to control infection |
| I found the labs really interesting as I not only got to see how easy it is to contract specific infections but more importantly discovered how easy it is to prevent the infections. |
| Initially questioned the relevance of labs to paramedic science, however once I actually saw the microbial growth it really changed my mind set - which used to be 'well I'm ok and healthy, so I don't need to worry too much about being exceptionally cautious'. But now I realise the extent to which my involvement as a potential carrier of pathogens will put my patient's at risk, and that it's not so much only about my health but the risk I am exposing to my patients. The lab's really helped to visualise the microbes that normally wouldn't be able to be seen day to day with the naked eye. |
| Actually being able to see the growth of microorganisms. I am a visual learner, and so microbiology sometimes goes over my head because I can't 'see' it. The labs really put the content into a visual canvas. |
